# Can Maintaining Optimal Magnesium Balance Reduce the Disease Severity of COVID-19 Patients?

**DOI:** 10.3389/fendo.2022.843152

**Published:** 2022-03-29

**Authors:** Mark Eskander, Mohammed S. Razzaque

**Affiliations:** Department of Pathology, Lake Erie College of Osteopathic Medicine, Erie, PA, United States

**Keywords:** magnesium, vitamin D, health, disease, hypomagnesemia

The coronavirus disease (COVID-19) caught the world by surprise, claiming millions of lives due to its deadly effects. Ongoing research studies evaluate the measures that can reduce the severity of symptoms in patients infected by the severe acute respiratory syndrome coronavirus 2 (SARS−CoV−2). Magnesium is an essential nutrient that has many studied benefits in humans. This brief commentary aims to describe the potential benefits of magnesium on COVID-19 patients and the reported effects of low versus high magnesium levels in SARS−CoV−2 infected individuals. Also, the potential benefits of vitamin D and how magnesium acts as a cofactor to activate vitamin D functions are elaborated. The results of the existing studies point towards evidence that magnesium may have significant benefits in reducing the severity of COVID-19 symptoms. There is also evidence that magnesium-dependent vitamin D activities may have antiviral effects, thus potentially being able to reduce rates of COVID-19 infection, which is a hypothesis that should be further tested.

The COVID-19 primarily causes respiratory distress but has a broad range of other clinical manifestations and affects various organs and systems in the body. COVID-19 is especially lethal to elderly populations at higher risk (1, 2). As of September 3rd, 2021, the current worldwide death rate of individuals infected by COVID-19 is 4,539,723 (World Health Organization). Many public health measures have already been taken to try to prevent the spread of COVID-19, such as face mask mandates, vaccine inoculation, quarantine, social distancing, limited capacities at public venues, and a shift to online work. However, COVID-19 is still proving to be a health concern as cases of infection continue to rise. The continued infection of individuals by COVID-19 may be due to the high transmissibility of the virus (3–6). It is also proposed that some of the new variants of the virus are even more rapidly transmissible and lethal. Thus, certain preventative measures are essential to find to reduce the severity of symptoms and improve patient outcomes in individuals that have been infected, since the prevention of infection is not always possible.

Basic nutrients that optimize physiologic functions in the body, such as magnesium, may be used as a prophylactic measure that can improve patient outcomes in individuals infected by SARS−CoV−2, and potentially even reduce the intensity of the infection by the virus by potentiating vitamin D functions. In the U.S., more than 50% of people are magnesium deficient (7, 8), and magnesium deficiency is also prevalent in many other countries. The low level of magnesium in the general population may make individuals more vulnerable to viral insults. The high prevalence of magnesium deficiency makes it important to determine how optimal and suboptimal magnesium levels affect COVID-19 patient outcomes, which this paper aims to elaborate on.

## Role of Magnesium in Health and Disease

**Figure 1:**
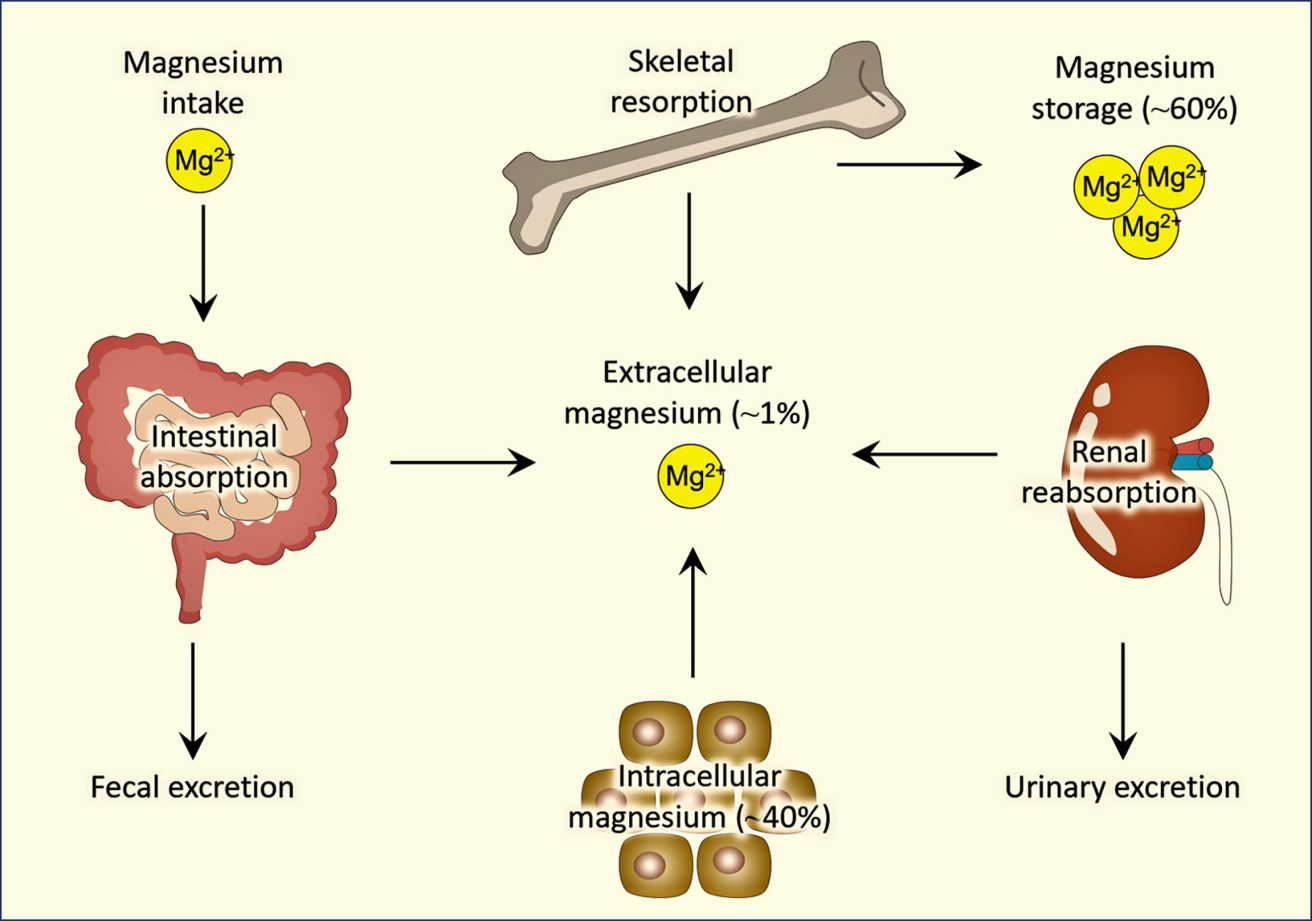
Simplified diagram showing regulation of magnesium homeostasis.

Magnesium is an essential nutrient required for many different physiologic functions in the body (9, 10). Magnesium homeostasis is mostly maintained by the cross-talks among intestine, bone, kidneys (**Figure 1**). The recommended daily amount of magnesium intake for 14-18 years is 360 mg (females)/410 mg (males), for 19-30 years is 310 mg (females)/400 mg (males), for people 31 years of age and older is 320 mg (females)/420 mg (males). Magnesium is a cofactor for over 600 enzymes in the body with diverse functions that play roles in many different systems (9, 11). One such system is the inflammatory system. Magnesium has been shown to have potent anti-inflammatory effects: low magnesium (0.14 ± 0.02 mmol) levels in rats have been associated with augmented inflammation (12), and higher magnesium levels have been associated with a reduction of C-reactive protein (CRP) levels (13, 14), which is one of the widely used biomarkers to measure inflammation, suggesting that magnesium has the potential to reduce inflammation. This has proven to be true with asthma patients (a disease with lung inflammation); the use of intravenous magnesium sulfate (MgSO_4_) and high-dose continuous MgSO_4_ infusion were both shown to reduce the odds of hospitalization in pediatric asthma patients (15, 16). Additionally, the use of nebulized MgSO_4_ in pediatric asthma patients (17) resulted in a significant reduction in the Yung Asthma Severity Score in pediatric asthma patients compared to pediatric asthma patients treated with a placebo (18). Separate studies showed improved outcomes of asthma patients of all ages when treated with nebulized MgSO_4_ (15–17). Thus, a wide record of evidence shows the efficacy of different forms of magnesium in reducing the intensity of asthma symptoms in patients of all age groups. This may implicate the clinical benefits of using magnesium in treating lung inflammation of patients with COVID-19. Different forms of magnesium have different levels of bioavailability. Studies show that organic magnesium, such as magnesium citrate, has higher bioavailability than inorganic magnesium, such as magnesium oxide (7, 19).

**Figure 2:**
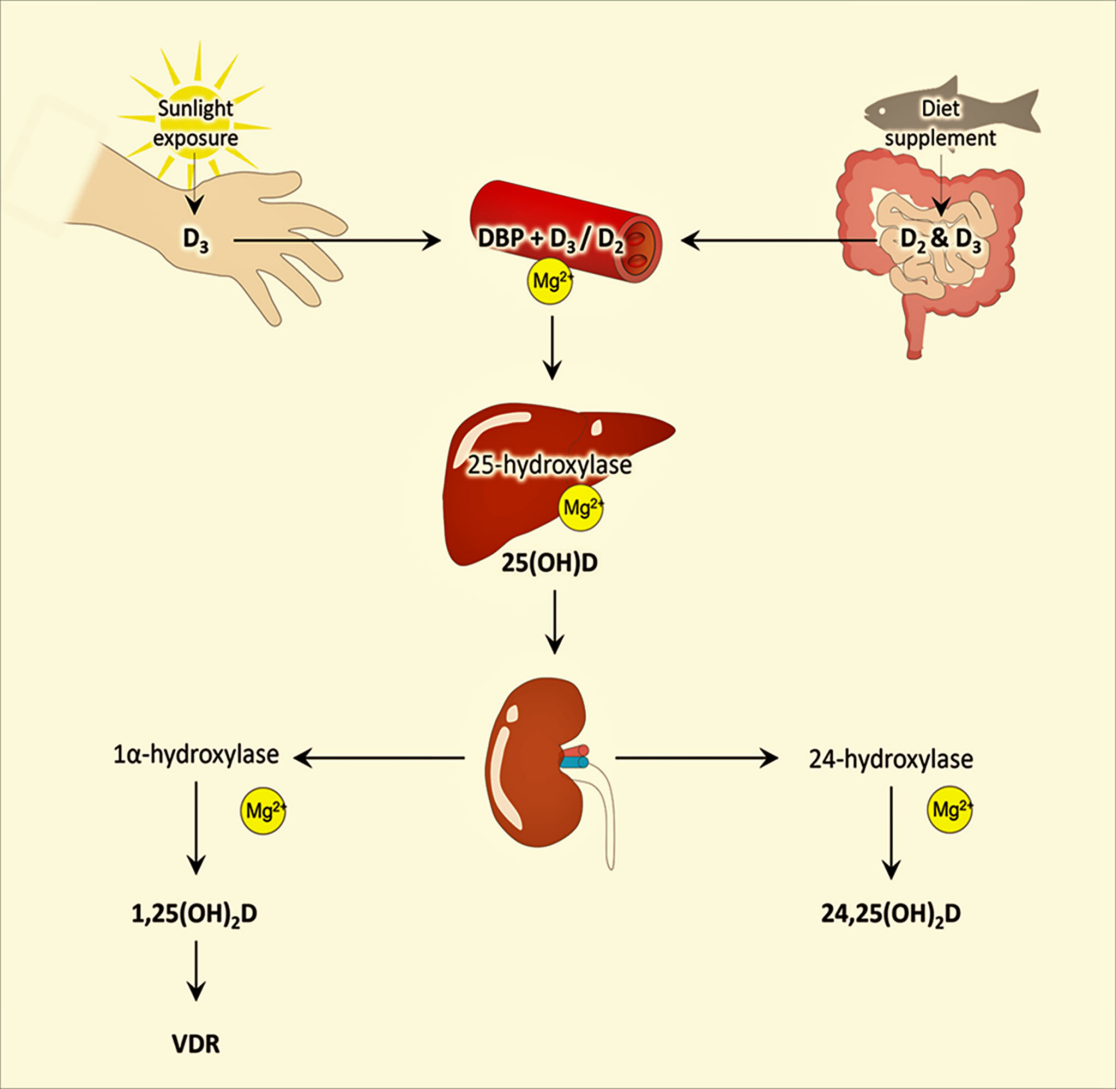
Simplified diagram of the different stages of vitamin D synthesis and role of magnesium in the activation of vitamin D; modified from earlier publications. For simplicity, only the essential steps of vitamin synthesis are included. VDR, vitamin D receptor; DBP, vitamin D binding protein.

Another essential role that magnesium plays in the body is its effect on activating vitamin D (11, 20, 21). The enzymes are required to convert the inactive form of vitamin D to the active form of vitamin D where magnesium acts as a cofactor (7, 11) (**Figure 2**). Studies have found the association of vitamin D in improving immune functions (22, 23), cell proliferation and organ regeneration (24), and reducing cardiovascular disease burden (25), as well as the widely accepted role of vitamin D in vascular, oral and bone health along with calcium metabolism (26–30). These health benefits of vitamin D will not be achieved even with adequate vitamin D levels if magnesium is deficient, thus emphasizing the essential role of maintaining optimal magnesium levels to attain desirable benefits of vitamin D. Magnesium deficiency is associated with cardiovascular diseases (31), hypertension (32), osteoporosis (33, 34), and diabetes (35).

## Vitamin D-Dependent Effects of Magnesium on COVID-19 Outcomes

In searching for the crucial need to reduce the deadly effects of COVID-19, maintaining optimal magnesium levels is an option that should be considered, as it has numerous protective roles in the body that may mitigate the deadly effects of COVID-19 (36). One such role that has been discussed is the activation of vitamin D *via* magnesium, and vitamin D’s essential role in improving immune functions (22, 23). Research on vitamin D and the immune system shows that there is a correlation between low vitamin D levels and autoimmune diseases, such as multiple sclerosis, rheumatoid arthritis, diabetes mellitus, and inflammatory bowel diseases (37–39). Additionally, several studies have shown that vitamin D has effects in reducing inflammatory cytokines and increasing anti-inflammatory cytokines (40, 41), indicating a potential clinical use of vitamin D in treating diseases that cause excessive inflammation, such as COVID-19. A key mechanism by which vitamin D reduces inflammatory cytokine is by upregulating T regulatory lymphocytes. Magnesium’s role in activating vitamin D leads to improved immune function, which can reduce the severity of the cytokine storm in COVID-19 infection (42). The cytokine storm requires a considerable amount of energy and leads to a loss of ATP. Magnesium and phosphate are required for the regeneration of ATP, thus magnesium can help replenish energy stores in the body to restore and boost immune system functions (43).

Recently published articles discussed several possible mechanisms in which vitamin D can help reduce SARS−CoV−2 infections and lessen the mortality of those affected patients (44). One mechanism discussed in which vitamin D can reduce viral infections is by maintaining tight junctions (45, 46), which may reduce viral infections because there is evidence that viral entry into cells may be due to viruses compromising the integrity of tight junctions (47). Another study showed that vitamin D receptor-deficient mice significantly reduced the expression of tight junction proteins specifically in the lungs compared to wild-type mice (48), making the role of vitamin D in reducing SARS−CoV−2 infection relevant since COVID-19 also affects the lungs. The studies show evidence of vitamin D preventing certain viral infections, which is consistent with the hypothesis that vitamin D plays a role in controlling viral infection by maintaining the integrity of tight junctions. One study showed that individuals who were supplemented with 4000 IU/day vitamin D for 10 days were less vulnerable to dengue virus infection than individuals who were supplemented with 1000 IU/day of vitamin D for 10 days (49). Additionally, data shows that vitamin D deficiency is associated with patients who have acquired certain viral infections, including some respiratory tract infections (50, 51).

In a retrospective cohort study conducted in Singapore General Hospital from January 15 to April 15, 2020, patients treated with a combination of vitamin D, magnesium, and vitamin B12 (DMB) showed improved clinical outcomes compared to patients that were not treated with DMB (52). This study highlights the indirect benefit magnesium may have on COVID-19 patients by activating vitamin D. A total of 43 patients all over 50 years of age were analyzed in this study, 17 of which received the DMB treatment, and 26 patients did not receive the treatment. The investigators analyzed patient data to determine if DMB treatment would reduce the need for oxygen therapy and/or intensive care support. The results showed that significantly fewer patients in the DMB group needed oxygen therapy during their treatment compared to the control group (3 of 17 vs 16 of 26, P= 0.006). The data also showed that 1 out of the 17 patients in the DMB group required ICU support compared to 8 out of the 26 patients in the control group (52). This study highlights the importance of magnesium, as in the absence of magnesium, vitamin D will not be activated to play a protective and preventive role against the SARS−CoV−2 virus.

## Low Magnesium and COVID-19

The role of magnesium in reducing asthma (lung inflammation) symptoms that was previously mentioned is key evidence that shows how magnesium may reduce COVID-19 symptoms. Since previous studies show that magnesium can reduce symptoms of asthma (15–18) and diminish inflammation (12–14), it is plausible that magnesium may help reduce the severity of COVID-19 symptoms by reducing lung inflammation. Additionally, magnesium deficiency has been associated with increased IL-6, a proinflammatory cytokine and a likely target for COVID-19 therapies (53).

Recent research suggests that magnesium does have protective effects against COVID-19 symptoms (**Table 1**). One retrospective cohort study analyzed essential and toxic metals levels in blood samples of 306 COVID-19 patients admitted to Tongji hospital in Wuhan, China (54). The patients were hospitalized for a mean of 30 days and were categorized by severity of disease according to Guidelines of the Diagnosis and Treatment of New Coronavirus Pneumonia published by the National Health Commission of China. The results of the study showed lower magnesium levels in patients with more severe COVID-19 symptoms. The median magnesium level in patients with severe COVID-19 symptoms was 38.33 mg/L, while the median magnesium level in the non-severe symptoms group was 39.46 mg/L, yielding a P value of 0.002 between the two groups. This study indicates that higher levels of magnesium may be protective against severe COVID-19 symptoms. Another retrospective study analyzed 396 COVID-19 patients who survived and 63 patients who did not survive in order to determine prognostic factors associated with death (55). The study was performed from January 30th to April 5th, 2020, and studied patients admitted to Shahid Modarres Hospital in Tehran, Iran. Among many other factors, blood magnesium levels of each patient upon admission to the hospital were studied. In the group of patients that survived, the average magnesium level was 1.83 ± 0.24 me/L compared to 1.61 ± 0.19 me/L in the group of patients that did not survive, yielding a P-value of < 0.0001 (normal magnesium range is 1.5-2.5 me/L). Thus, this study also indicates that magnesium might have a protective role against COVID-19.

**Table 1:** Published studies with magnesium status in patients with COVID-19.

Investigators	Number of patients studied	Results
Zeng et al. (54)	306	COVID-19 patients with lower magnesium levels had more severe symptoms.
Alamdari et al. (55)	396	COVID-19 patients with higher magnesium levels had lower mortality rates.
Gunay et al. (56)	629	COVID-19 patients with lower magnesium levels had a higher degree of myocardial damage.
Zhu et al. (57)	83	Hypomagnesemia was more prevalent in COVID-19 patients who did not survive.
Beigmohammadi et al. (58)	60	Lower magnesium levels in COVID-19 patients correlated to higher disease severity and risk of mortality.
Pulido-Perez et al. (59)	118	Lower magnesium levels were associated with increased mortality.
Quilliot et al. (60)	300	Hypomagnesemia was associated with 61% of all patients in study. Moderate cases were associated with hypomagnesemia; critical cases were associated with higher magnesium levels.
Sharma et al. (61)	193	Severity of the disease was greater in patients with hypermagnesemia.

A retrospective cohort study done on 629 COVID-19 patients admitted to the hospital aimed to find a relationship between serum magnesium levels and myocardial damage and prognosis of disease (56). Serum troponin levels above the 99th percentile upper reference limit (24 - 30 pg/mL) was defined as myocardial damage. Prognosis of disease was determined by survival, thus the two groups in this study were the survival group and non-survival group. The median blood magnesium levels of the non-survival group were 1.94 mg/dl and in the survival group it was 2.03 mg/dl (P = 0.027) (normal range of serum magnesium = 1.7 - 2.5 mg/dL). The median troponin levels in the non-survival group were 25.2 pg/ml and in the survival group it was 4.5 pg/ml (P <0.001). The non-survival group’s median troponin levels were above the 99th percentile upper reference limit range, thus they were defined as having myocardial damage, while the survival group did not. Therefore, this study uncovered a significant correlation between lower magnesium levels and higher degree of myocardial damage in COVID-19 patients (56).

Another retrospective cohort study aimed to determine the significance of low magnesium levels in COVID-19 patients (57). 83 patients who were hospitalized in the Guanggu Hospital District, Wuhan Third Hospital, China were studied. The serum magnesium levels of the patients were studied, and the patients were separated into different groups based on disease severity (moderate, severe, critical), which was classified according to the fifth edition of China’s guidelines. The incidence of hypomagnesemia in each group of severity were as follows: 12.50% in the moderate group, 3.85% in the severe group, and 43.75% in the critical group. The results showed statistical significance for hypomagnesemia being more prevalent in the critical group than in the moderate and severe groups (P < 0.05). The results also showed that hypomagnesemia was more prevalent in non-survivors (40%) than in survivors (17.65%) (P < 0.05).

One cross-sectional study analyzing 60 COVID-19 patients admitted to the Intensive Care Unit (ICU) of Imam Khomeini Hospital in Iran between March and June 2020 focused on the association of serum level of micronutrients with the severity of COVID-19 disease (58). The severity of disease was measured with the Acute Physiology and Chronic Health Evaluation (APACHE) scoring system, where a score of ≥25 was consistent with high risk of mortality. Of the 60 patients studied, there were 20 patients with an APACHE score ≥25 and 40 patients with an APACHE score <25. It was found that levels of serum vitamin D, zinc, and magnesium were each significantly lower in the group with an APACHE score ≥25 (P < 0.001).

A retrospective study on 118 patients studied the relationship of renal function and serum magnesium levels on COVID-19 patients with type 2 diabetes (59). The patients were split into two groups: one group included patients with type 2 diabetes and the other included patients without type 2 diabetes. Renal function was measured by the patient’s estimated glomerular filtration rate (eGFR). Patients with type 2 diabetes had a lower eGFR than patients without type 2 diabetes, indicating a reduced renal function in the COVID-19 patients with type 2 diabetes (59.7 ± 32.8 vs. 78.4 ± 33.8 mL/min per 1.73 m^2^) (P = 0.008). Additionally, Patients with type 2 diabetes had lower serum magnesium levels than patients without type 2 diabetes (1.9 ± 0.3 vs. 2.1 ± 0.3 mEq/L) (P = 0.012). Statistical analysis of the patient outcomes showed that the type 2 diabetes group was associated with significantly increased risk of death, meaning that lower eGFR and magnesium levels were associated with increased mortality (59).

## High Magnesium and COVID-19

A prospective cohort study analyzed the serum magnesium levels of 300 patients in Nancy Brabois University Hospital between March 1, 2020, and April 29, 2020, and each patient was graded for COVID-19 severity according to WHO guidelines (60). The study showed a high prevalence of low magnesium levels in hospitalized COVID-19 patients; about 61% of the 300 patients in the study presented with hypomagnesemia (<0.75 mmol/L). However, this study revealed that most of the patients with critical cases had high magnesium levels, and most of the patients with moderate cases had low magnesium levels. The results of this study showed that the prevalence of hypomagnesemia (<0.75 mmol/L) was significantly higher in the group of patients who had a moderate case of COVID-19 (average Mg: 0.73 mmol/L) compared to the group of patients who had a critical case of COVID-19 (average Mg: 0.79 mmol/L) (P<0.001). The group of patients with critical cases of COVID-19 had the highest magnesium levels compared to the moderate and severe groups.

Although this study found that magnesium levels tend to be lower in patients with COVID-19 infection, one study that focused on pregnant women infected with COVID-19 found that pregnant women infected with COVID-19 had higher serum magnesium levels (62). This study was a systematic review of 385 pregnant women from 33 studies with COVID-19. 95.6% of the infected women in this study had a mild case of COVID-19, while 3.6% had a severe case, and 0.8% had a critical case. The study compared certain element levels, including magnesium, in pregnant women with COVID-19 infection and without infection as a control group. The results showed that pregnant women with COVID-19 infection in their first and third trimester had significantly higher magnesium levels than in the control group (first trimester: 1,557 ± 0,211 vs 1,848 ± 0,335, P < 0,0001) (third trimester: 1,947 ± 0,657 vs 2,767 ± 0,394, P < 0,0001). However, there was no correlation between magnesium levels and the severity of disease found in this study. The investigators could not find a definitive reason for this rise in magnesium levels seen in pregnant women infected by COVID-19.

In addition to previous studies mentioned, critical COVID-19 cases were also found to be associated with hypermagnesemia in a retrospective cohort study of 193 COVID-19 patients in a medical center in California conducted from March 13, 2020, to February 2, 2021 (61). The authors of the study hypothesized that hypermagnesemia would be a predictor of mortality and morbidity in COVID-19 patients because hypermagnesemia is associated with mortality in other critical illnesses. Patients were separated according to their magnesium levels upon admission to the hospital: 104 patients were in the hypermagnesemia group (Mg level ≥2.5 mg/dL) and 89 patients were in the normomagnesemia group (Mg level 1.7 - 2.5mg/dL). The outcomes and biomarkers of each group were compared. The severity of disease was greater in the hypermagnesemia group, indicated by 48% of patients with hypermagnesemia being admitted into the ICU versus only 15% of patients in the normomagnesemia group (P<0.001), the average duration of hospital stay was 15.42 days in the hypermagnesemia group compared to 6.7 days in normomagnesemia group (P=0.0001), and of the 35 patients in the study who required a ventilator, 34 of them were in the hypermagnesemia group (P<0.0001). Cough (P=0.005) and dyspnea (P=0.001) were also significantly more prevalent in the hypermagnesemia group compared to the normomagnesemia group. The electrolyte imbalance and mineral ion dysregulation are usually encountered in patients with ventilator (critical stages of the disease process), which is likely to be the consequence of multi-organ dysfunctions.

These results may be due to the adverse effects of hypermagnesemia. Of clinical relevance, hypermagnesemia has been shown to cause adverse cardiovascular, neurological, and respiratory effects (63). Since there are various units for measuring magnesium that are commonly used, errors in administering proper doses of magnesium are a common cause of its overdoses. Two case reports regarding the treatment of two different alcoholic withdrawal patients highlight this issue along with the symptoms associated with magnesium overdose (64). In the course of these patients’ treatment, they were ordered to be administered 2 g of magnesium sulfate intravenously. However, patients were given 20 g of magnesium sulfate by mistake leading to magnesium overdose, leading to cardiac arrest in both the patients, followed by successful resuscitation (64). Adverse cardiac effects of magnesium overdose include delays in conduction of electrical impulses in the heart, asystole (cardiac arrest). Other adverse effects of magnesium overdose include apnea, coma, neurologic deterioration, and muscle weakness.

## Conclusions

There is strong evidence of magnesium playing a role in reducing the severity of asthma symptoms (15–18), COVID-19 symptoms (54–59), and inflammation (12–14). Magnesium has been shown to exert anti-inflammatory effects both independently and as a result of activating vitamin D (11–14, 20, 21, 53). The anti-inflammatory effects of magnesium are potentially responsible for reducing symptoms of asthma patients treated with magnesium. Adequate magnesium levels have been associated with lower mortality rates in COVID-19 patients and less severe symptoms (52, 54–59). Natural sources of magnesium (avocado, spinach, almonds, pumpkin seeds, whole grains, black beans, wheat, and oatmeal) and magnesium supplementation are easily accessible and affordable that can be consumed to reduce the disease burden of COVID-19 patients. Magnesium’s role in activating vitamin D is essential in that vitamin D has been shown to reduce the rate of viral infection in patients with several respiratory tract infections. Thus, more research should be conducted to determine the magnesium-dependent effects of vitamin D in reducing the risk of infection in patients with COVID-19.

## Author Contributions

ME collected information and drafted the manuscript. MR conceptualized and reviewed the manuscript. Both authors contributed to the article and approved the submitted version.

## Conflict of Interest

The authors declare that the research was conducted in the absence of any commercial or financial relationships that could be construed as a potential conflict of interest.

## Publisher’s Note

All claims expressed in this article are solely those of the authors and do not necessarily represent those of their affiliated organizations, or those of the publisher, the editors and the reviewers. Any product that may be evaluated in this article, or claim that may be made by its manufacturer, is not guaranteed or endorsed by the publisher.
